# A modeling study of the impact of photolysis on indoor air quality

**DOI:** 10.1111/ina.13054

**Published:** 2022-06-13

**Authors:** Zixu Wang, David Shaw, Tara Kahan, Coralie Schoemaecker, Nicola Carslaw

**Affiliations:** ^1^ Department of Environment and Geography University of York York UK; ^2^ Department of Chemistry University of Saskatchewan Saskatoon Saskatchewan Canada; ^3^ CNRS, UMR 8522 – PC2A – Physicochimie des Processus de Combustion et de l’Atmosphère Université Lille Lille France

**Keywords:** artificial lights, attenuated sunlight, hydroxyl radicals, indoor air chemistry, indoor air model, indoor photolysis

## Abstract

The importance of photolysis as an initiator of air chemistry outdoors is widely recognized, but its role in chemical processing indoors is often ignored. This paper uses recent experimental data to modify a detailed chemical model, using it to investigate the impacts of glass type, artificial indoor lighting, cloudiness, time of year and latitude on indoor photolysis rates and hence indoor air chemistry. Switching from an LED to an uncovered fluorescent tube light increased predicted indoor hydroxyl radical concentrations by ~13%. However, moving from glass that transmitted outdoor light at wavelengths above 380 nm to one that transmitted sunlight above 315 nm led to an increase in predicted hydroxyl radicals of more than 400%. For our studied species, including ozone, nitrogen oxides, nitrous acid, formaldehyde, and hydroxyl radicals, the latter were most sensitive to changes in indoor photolysis rates. Concentrations of nitrogen dioxide and formaldehyde were largely invariant, with exchange with outdoors and internal deposition controlling their indoor concentrations. Modern lights such as LEDs, together with low transmission glasses, will likely reduce the effects of photolysis indoors and the production of potentially harmful species. Research is needed on the health effects of different indoor air mixtures to confirm this conclusion.

## 1 INTRODUCTION

In developed countries, people spend most of their time (~90%) indoors,^1^, ^2^ where they consequently receive most of their exposure to air pollution. The ongoing COVID‐19 pandemic has heightened awareness of the importance of good indoor air quality. Many governments have asked their citizens to work or study at home, and restricted travel to prevent the spread of disease.^3^ Therefore, air quality in the indoor environment and especially in our homes has become more important than ever.

The role of photolysis as a mediator of atmospheric chemistry has long been recognized for the ambient atmosphere, but there has been far less focus on the role that photolysis can play indoors.^4^ Indoor light includes artificial lighting indoors and attenuated sunlight that can move into indoor environments through windows and skylights. Nazaroff and Cass^5^ were the first to recognize the importance of indoor photolysis, using a simple model to show that increased photolysis rates enhanced the rate of chemical reactions, producing higher concentrations of reactive species. Carslaw^6^ investigated the indoor air chemistry of a typical urban residence in the UK with a detailed chemical box model, showing that light intensity level indoors were a key determinant of model uncertainty when simulating OH concentrations. The simulated indoor OH concentration was ~4 × 10^5^ molecule/cm^3^ assuming that 3% and 10% of outdoor UV and visible light were transmitted indoors, respectively, but this concentration increased by 281% when UV and visible transmission increased to 27.5% and 75%, respectively. These higher simulated OH concentrations were confirmed by the measurements of Gomez Alvarez et al.^7^, who measured up to 1.8 × 10^6^ molecule/cm^3^ of OH in a school classroom in Marseille, when light shone directly through a window and photolysed nitrous acid (HONO) to produce OH.

The contribution of artificial light to overall photolysis indoors depends on the location of the light within the room, the geometry of the room, and the type of light, with different artificial lights having unique spectral^8^ and spatial^9^ characteristics. The amount of light that can penetrate indoors from outdoors is influenced by the type of window, time of year and day, the building orientation and location and meteorological conditions (e.g., cloudiness). For instance, Crawford^10^ found that an unoccluded solar disk with slightly overcast conditions enhanced spectral actinic flux by 20% compared to clear sky conditions, while an 80% reduction was noted for more overcast conditions. Blocquet et al.^11^ used both modeling and measurements to investigate the spatial and spectral distribution of sunlight which passed from outdoors through windows, finding that 0.15% to 30% of outdoor UV light (300–400 nm) and 0.7% to 80% of outdoor visible light (400–750 nm) were observed indoors depending on the glass type and time of day.^11^ Similar reductions were reported by Zhou et al.^4^ for 77 windows and glass samples.

It is worth considering how lighting and glazing has changed in recent years. The long lifetimes and high efficiency characteristics of fluorescent tubes (used mainly in office blocks and industrial settings) led to their being a dominant indoor lighting source for many years in such locations.^12^ However, LED lights are becoming more popular, owing to much higher energy efficiency compared to more traditional lighting. For instance, they are estimated to provide 56%–62% energy savings and an increase in lifetime by a factor of 9 compared to the use of fluorescent tubes.^13^, ^14^ In residential settings, incandescent lighting was a dominant lighting source for many years.^15^ However, this type of lighting is also being replaced by LED lights. Relative to incandescent lights, LEDs use ~85% less energy and have 50 times longer lifetimes^16^ and are likely to remain as the dominant source of illumination in the future.^17^

Glass composition has also become increasingly sophisticated in recent years, such as through multipane glazing,^18^ tinting,^19^ low‐emissivity coatings,^20^ anti‐reflective coatings,^21^ and vacuum glazing,^22^ compared to the single pane and compositionally simple glass types that used to be more common.^23^ These changes will undoubtedly affect levels of indoor lighting and hence indoor air chemistry.

There have been a few papers that have focused on the impacts of different drivers of indoor air chemistry to date. For instance, Zhou and Kahan^24^ undertook a thorough photochemical characterization of a test house in Texas, including the determination of spatial and temporal photochemical rate constants and quantification of the effects of cloud cover and diffuse light. In addition, Zhou et al.^4^ investigated the impacts of different window materials and outdoor meteorological conditions on indoor photolysis rates. However, detailed chemical models can provide deeper insight and consider a wider range of conditions than experimental data alone. This paper describes an improved representation of photolysis in a detailed chemical model for indoor air. The improved model is then used to investigate the impacts of different controlling factors on indoor photolysis rates and hence indoor air chemistry.

## 2 METHODS

### 2.1 The INDCM model

The model used in this study is the INDCM (INdoor air Detailed Chemical box Model), which was developed by Carslaw^6^ and improved by Carslaw et al.^25^ The basis of the INdoor air Detailed Chemical box Model (INDCM) is a comprehensive chemical mechanism (the Master Chemical Mechanism, MCM v3.2, http://mcm.york.ac.uk/), that includes around 20,000 reactions and 5000 species, and represents the near‐explicit degradation of ~143 VOCs in the gas‐phase.^26^, ^27^, ^28^, ^29^ The chemical degradation of each VOC is initiated by reaction with hydroxyl (OH) radicals, nitrate radicals (NO_3_), ozone (O_3_), and/or photolysis where relevant. Radicals are generated through the first oxidation step, including RO (oxy), RO_2_ (peroxy), and RRCOO (Criegee) radicals, which can each undergo a number of further reactions until the final oxidation products of CO_2_ and H_2_O are formed.^27^ The model also includes terms that represent deposition to and emission from surfaces, exchange with outdoors and gas‐to‐particle partitioning reactions for limonene.^25^

### 2.2 Representation of artificial lighting

The INDCM considers 35 photolysis processes for either individual or groups of species based on the Master Chemical Mechanism protocol.^27^, ^29^ For these 35 processes, 27 occur in the UV region only, 5 in the UV and visible regions and 1 in the visible only (see Table S1). In the previous version of the model,^6^ flat transmission of light in the UV and visible wavelength ranges was assumed from outdoors, with only one type of indoor lighting, based on the methods described in Nazaroff and Cass.^5^

The photolysis coefficient (*j*) for each species *i*, was calculated using

(1) $$j_{i}=h_{\text{uv}}\left(\lambda_{300}^{400}\right)I_{\text{uv}}\left(\lambda_{300}^{400}\right)+h_{\text{vis}}\left(\lambda_{400}^{760}\right)I_{\text{vis}}\left(\lambda_{400}^{760}\right)$$

where

$$h_{\text{uv}}\left(\lambda_{300}^{400}\right)={\left(100\;\text{nm}\right)}^{‐1}\int_{300\;\text{nm}}^{400\;\text{nm}}\sigma \psi \;d\lambda$$

$$h_{\text{vis}}\left(\lambda_{400}^{760}\right)={\left(360\;\text{nm}\right)}^{‐1}\int_{400\;\text{nm}}^{760\;\text{nm}}\sigma \psi \;d\lambda$$

In the above equations, *I*_uv_ and *I*_vis_ represent the spherically integrated photon fluxes (photons/cm^2^/s) in the UV and visible bands, and were assumed to have values of 2.3 × 10^13^ and 7.0 × 10^14^ photons/cm^2^/s, respectively.^5^ *h*_uv_ and *h*_vis_ are calculated as the integral of the absorption cross‐section (σ, cm^2^) and the quantum yield (ψ) as a function of the wavelength (λ, nm). These values were calculated using the relevant literature such as IUPAC or the MCM protocol^27^, ^29^ for each individual photolysis process.

For the modifications made for this study, the UV wavelength region from 300 to 400 nm was split into ten different 10 nm sub‐regions (300–310 nm; 310–320 nm, etc.). In the 400–800 nm wavelength region, fewer species absorb and transmission is much flatter than in the 300–400 nm wavelength range,^8^ so it was considered as one further wavelength interval.

The overall photolysis rate coefficient (*j*) was calculated for each species using a modified form of Equation 1 as

(2) $$j_{i}=\left(\sum_{x=0}^{9}h_{\text{uv}}\left(\lambda_{300+10x}^{310+10x}\right)I_{\text{uv}}\left(\lambda_{300+10x}^{310+10x}\right)\right)+h_{\text{vis}}\left(\lambda_{400}^{800}\right)I_{\text{vis}}\left(\lambda_{400}^{800}\right)$$

Equation 2 was used to calculate new photolysis coefficients for the 35 photolysis processes and for 7 different indoor artificial lights (Incandescent, Halogen, LED, compact fluorescent lamps (CFL), covered or uncovered fluorescent tubes (CFT/UFT), and fluorescent tubes (FT)) based on the spherically integrated photon fluxes adjacent to these lights measured by Kowal et al.^8^ Note that FT was a new fluorescent tube used only during the experiment, while CFT and UFT were pairs of fluorescent tubes mounted in (covered or uncovered) ceiling fixtures. The values used in the calculation of *h*_uv_ and *h*_vis_ (σ,ψ) were taken from IUPAC or the MCM protocol.^27^, ^29^

Kowal et al.^8^ reported the calculated photolysis rate coefficients for hydrogen peroxide (H_2_O_2_), O_3_, nitrous acid (HONO), formaldehyde (HCHO), and acetaldehyde both adjacent to and 1m away from different light sources. This permitted the % of light at 1 m relative to that adjacent to the indoor lights to be calculated. The transmission of light at 1 m distance was assumed to be 2% of the adjacent value for all species for LED, halogen, incandescent, and CFL lights, 4% for all species for FT light and 15% for all species with CFT and UFT lights. Different distance dependencies have been ascribed to C(U)FT deviating further from a point source than FT and to scattering from the light fixtures. The value at 1 m can be considered to be more representative for an integrated average for a room.^8^ The calculated values at 1m for the 7 light sources for the 35 processes are presented in Table S2.

### 2.3 Representation of attenuated outdoor sunlight

The previous model assumed that 3% of UV and 10% of visible light from outdoors passed through the windows and ended up indoors.^6^ However, in reality, transmission of outdoor light indoors will vary depending on the window material (glass) composition. Blocquet et al.^11^ measured or reported from previous studies, the transmittance of light through 17 different windows. For this work, three different glasses were selected from their study, that encompassed a wide range of cutoff wavelengths (at the lower end of transmission), including “Glass C Sacht Self‐cleaning” (transmission from 315 to 800 nm, [Glass C],^30^), “Low Emissivity” (transmission from 330–800 nm, [LE]), and “Low Emissivity With Film” (transmission from 380 to 800 nm, [LEWF]).

For each glass, the % of transmitted light was defined over the relevant wavelength range for each absorbing species. The value of σψ for each photolysing individual or group of species was then calculated between 300 and 800 nm, using data from IUPAC^31^ or the MCM protocol^27^, ^29^ and the two sets of information combined to calculate weighted transmission factors for each wavelength interval. Finally, the contributions from each individual wavelength over the entire 300–800 nm wavelength range were summed to provide a single transmission factor for each photolysing species and for each window material. An example for this calculation is shown in Table S3, and transmission factors for all 35 photolysing species/groups of species and for the three window glasses based on this method are shown in Table S4.

### 2.4 Model simulations

The model location was set to York, UK, and the date was set to June 21. The indoor temperature was assumed to be 300 K, relative humidity was 45% and AER was 0.76 h^−1^, the latter based on the results of Murray and Burmaster.^32^ The outdoor concentrations of nitric oxide (NO), nitrogen dioxide (NO_2_), and O_3_ in the model varied diurnally based on background UK suburban concentrations as described in Carslaw,^6^ with average daily concentrations of 6.2, 15.0, and 22.9 ppb, respectively. Outdoor VOC concentrations and indoor VOC emission rates were initialized based on,^33^ as reported in Carslaw.^6^ In order to make sure that the model reaches steady state, it is set to run for three days and data from the third day are used for all of the analyses that are presented in the results section. Individual settings for indoor lights and glass material are described in each section of the results.

In terms of model outputs, we explored the concentrations of key radical species, OH, hydroperoxy (HO_2_) and organic peroxy radicals (RO_2_) as well as those of O_3_, HONO, formaldehyde (HCHO), NO_2_, NO and two important groups of secondary products produced through chemical reactions. These are the sum of the 234 peroxyacetyl nitrate species in the model (TOTPAN) and the sum of the 304 organic nitrates (TOTORGNO3). Previous studies have identified toxic impacts of PANs not only on animals and plants, but also on humans,^34^, ^35^ such as skin cancer,^36^ changes in the DNA bases,^37^ mutagenicity,^38^, ^39^ and eye irritation.^40^, ^41^ Organic nitrates were also found to have adverse health effects.^42^ These two groups of species therefore act as a proxy for the potentially harmful species that can be formed through secondary chemistry indoors under the different lighting conditions.

### 2.5 Spectral radiometer measurements

Photolysis values were measured indoors using a spectral radiometer, which provided a direct measurement of solar actinic UV flux and permitted the determination of photolysis frequencies.^43^ The instrument consists of a 2‐π steradian quartz diffuser coupled to an Ocean Optics spectrometer via a 10 m fiber optic cable. The spectrometer operates between 200 and 1000 nm and is calibrated over the wavelength region from 250 to 750 nm (<1 nm resolution). It utilizes a Hamamatsu, back‐thinned FFT‐CCD detector with more than 90% quantum efficiency at 700 nm. It has an integration time between 8 ms and 15 min and fully automated data collection using Spectrasuite software (NCAS, 2018).^44^

The measurements were made in a first floor office in the Environment building at the University of York for 10 days in January 2018 (January 20–January 29). The radiometer was placed on an office windowsill. For 9 of these days, the lights in the office were off. The results focus on 25th, which was the sunniest day with internal lights off.

## 3 RESULTS AND DISCUSSION

### 3.1 Impact of model improvements on predicted concentrations

The first test was to compare the impacts of these changes to the previous model output. Table 1 shows the differences between the daily average concentrations of the key output species for each of the 7 studied indoor artificial lights and the previous model representation that assumed indoor lighting was incandescent. For all these simulations, it was assumed there was no attenuated sunlight, so the differences reflect those in the representation of the artificial lighting only. These differences are most important for the radical species as expected, with maximum differences of ~40% and 60% for OH and HO_2,_ respectively, for UFT lighting compared to the old representation. For other species such as HCHO and NO_2_, the differences are minimal.

**TABLE 1:** Difference (%) between daily average concentrations of the key chemical species studied for 7 indoor artificial lights and the old model representation

	O_3_	HONO	HCHO	OH	HO_2_	RO_2_	NO	NO_2_
Incandescent	−0.9	0.1	0	−9.2	−15.1	−7.6	8.2	0.1
Halogen	−0.8	0.1	0	−9.0	−15.5	−8.2	8.9	0.1
LED	−1.4	0.3	0	−12.2	−16.5	−6.6	7.1	0.2
CFL	0.4	−0.4	0	−2.8	−16.4	−13.2	15.6	−0.3
UFT	4.5	−1.1	−0.1	39.8	58.0	21.8	−17.7	−0.8
CFT	−1.1	0.2	0	−11.0	−16.5	−7.7	8.3	0.1
FT	0.6	−0.1	0	4.6	5.3	1.5	−1.5	−0.1

Table 2 shows the % difference in average indoor concentrations for the same species for the three glasses (Glass C, LE, and LEWF) compared to the old representation that assumed 3% of UV and 10% of visible light were transmitted through the windows. For these simulations, we assumed no indoor lighting. Daily average concentrations for the former model representation are most similar to those in the new simulations for the LEWF glass. Differences between the old representation of attenuated sunlight and the new Glass C predicted concentrations are more than 400% and 170% for OH and NO, respectively. These results show that there can be a wide variation in predicted concentrations for some species, depending on the assumptions made around indoor photolysis conditions.

**TABLE 2:** Difference (%) between daily average concentrations of key chemical species for the 3 studied window materials and the old representation

	O_3_	HONO	HCHO	OH	HO_2_	RO_2_	NO	NO_2_
Glass C	64.8	−19.8	0.5	418.0	15.6	−26.3	171.2	−8.8
LE	34.7	−11.3	0.4	208.3	−2.3	−32.9	117.9	−5.6
LEWF	5.5	−1.2	0.1	29.3	−3.7	−21.4	22.8	−1.3

### 3.2 Comparison with measurement

**FIGURE 1:**
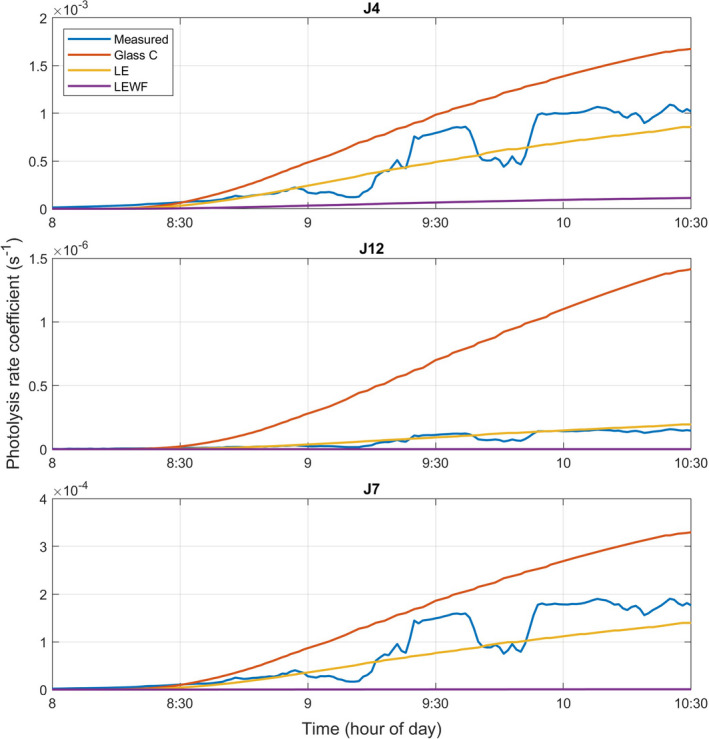
Profiles of J4 (NO_2_), J12 (HCHO to H_2_ and CO) and J7 (HONO) determined from measured irradiance from the spectral radiometer and from model results assuming Glass C, LE, and LEWF. The location and time of year were set as the University of York on January 25

Figure 1 shows the profiles of J4 (NO_2_), J12 (HCHO to H_2_ and CO), and J7 (HONO) determined from measured irradiance from the spectral radiometer and predicted by the new model parameterisations between 08:00 and 10:30 h on January 25, 2018, when the sun was shining directly into the office (and with no internal lighting). These profiles show that measurements were affected by clouds from time to time, but that the profiles are in reasonable agreement with the model results, particularly for LE. This was also the case for J5 (NO_3_ to NO and O_2_) and J6 (NO_3_ to NO_2_ and O^3^ (P)), which are not shown. Differences between the measured values and model predicted values were quantified through a root mean square difference calculation for each minute for which measured data were available. The results show that the measured *j* values are most similar to the simulated LE glass results (root mean square difference was 111%, 45%, and 100% of the mean measured value for Glass C, LE, and LEWF glass, respectively) and that the new model parameterization appears to be representative of light entering a room via a window.

### 3.3 Impacts of different indoor artificial lights on indoor air chemistry

Table 3 shows the average model simulated concentrations between 06:00 and 18:00 h (daylight hours) of key indoor species for the 7 different light sources assuming the same glass type (Glass C), with attenuated sunlight through Glass C with no indoor lighting and completely dark conditions indoors added for comparison. There is relatively little variation in the predicted concentrations of our selected indoor species when the indoor artificial light type changes, although there is evidence for significant chemical activity indoors compared to the dark conditions.

**TABLE 3:** Average concentrations between 06:00 and 18:00 h of key indoor species for the seven artificial lights assuming the same glass (Glass C) type (attenuated sunlight level is identical for each run) in rows 1–7, attenuated sunlight only for the same glass in row 8 and for darkness in row 9

	O_3_ (ppb)	HONO (ppt)	HCHO (ppb)	NO_2_ (ppb)	OH (10^5^ molecules/cm^3^)	HO_2_ (ppt)	RO_2_ (ppt)	NO (ppb)	TOTPAN (ppt)	TOTORGNO_3_ (ppt)
Incandescent	7.7	159.5	33.1	3.1	7.6	4.6	5.5	2.6	330.5	167.0
Halogen	7.7	159.5	33.1	3.1	7.6	4.6	5.5	2.6	330.1	167.0
LED	7.7	159.6	33.1	3.1	7.6	4.6	5.5	2.6	329.2	166.5
CFL	7.7	159.2	33.1	3.1	7.6	4.6	5.5	2.6	329.9	167.6
UFT	8.1	157.8	33.0	3.0	8.6	5.8	6.6	2.3	390.7	181.8
CFT	7.7	159.6	33.1	3.1	7.6	4.6	5.5	2.6	329.2	166.6
FT	7.8	159.1	33.1	3.0	7.9	4.9	5.8	2.5	347.2	171.3
Attenuated sunlight only	7.7	159.6	33.3	3.1	7.6	4.6	5.5	2.6	329.2	166.5
Dark	4.0	250.6	33.1	3.6	1.0	3.7	8.3	0.7	166.4	26.5

**FIGURE 2:**
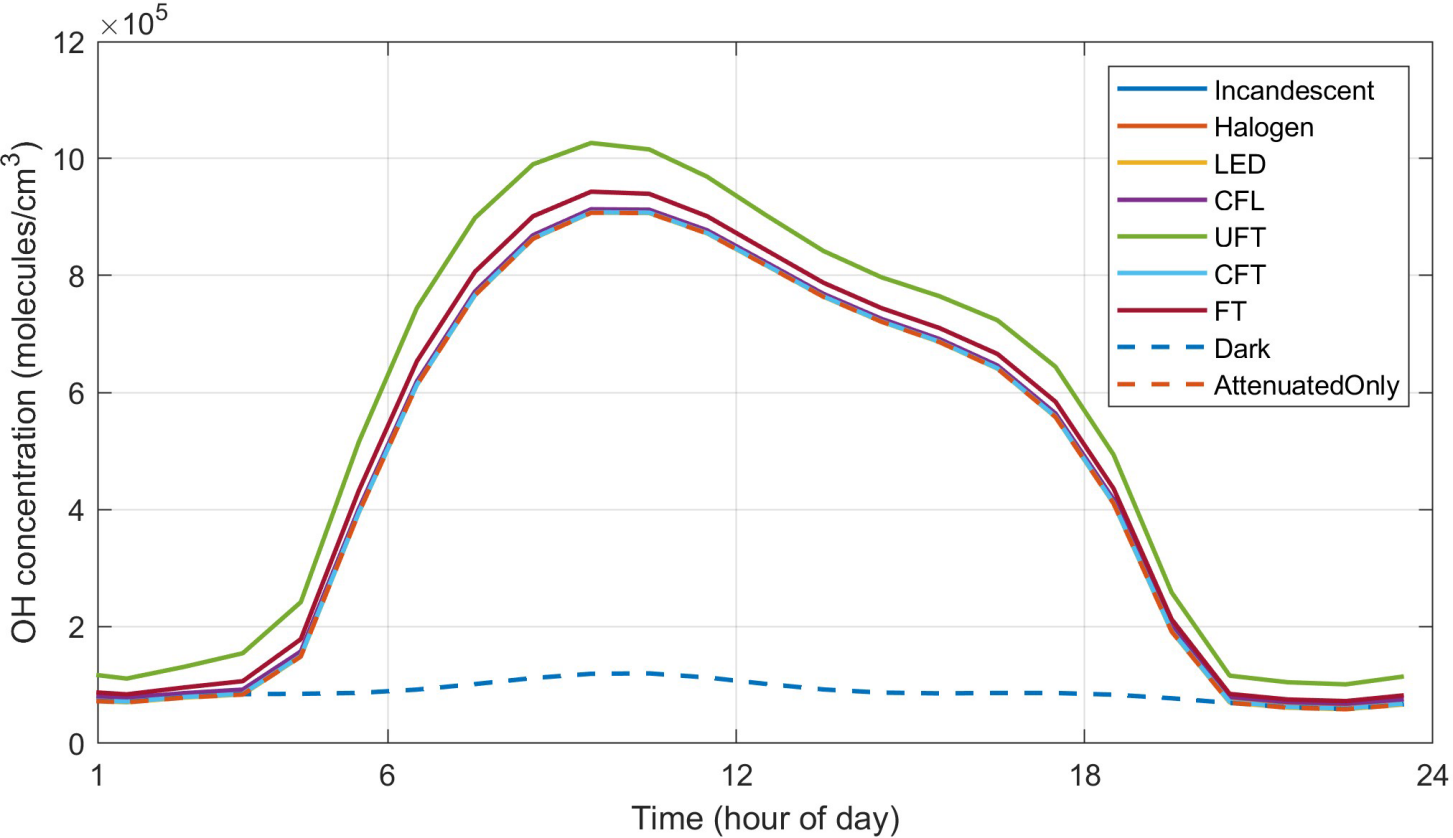
Hourly averaged concentrations of OH for different artificial lights (Incandescent, Halogen, LED, CFL, UFT, CFT, and FT) with attenuated sunlight through Glass C, with the dark and attenuated sunlight only simulations shown for comparison. The Incandescent, Halogen, LED, and attenuated sunlight only profiles are overlaid by the CFT plot

The highest OH (see Figure 2) and O_3_ values are for UFT lighting (peak values of 1.0 × 10^6^ molecule/cm^3^ and 9.5 ppb, respectively) and FT lighting (peak values of 9.4 × 10^5^ molecule/cm^3^ and 9.3 ppb, respectively). Although O_3_ is photolysed indoors, its production via NO_2_ photolysis more than outweighs the photolytic loss, so that overall, more indoor lighting increases ozone concentrations (and reduces NO_2_ concentrations). The NO produced from both photolysis of HONO and NO_2_ can react with HO_2_ to produce OH and also suppresses RO_2_ concentrations. These reactions lead to slightly lower peak NO concentrations for UFT and FT than the other lights.

HCHO concentrations are more or less invariant. The highest concentration is observed for attenuated sunlight, where the HCHO formed through secondary chemistry (e.g., from VOC oxidation) must outweigh any removal by direct photolysis. PANs and organic nitrates are formed through the reactions of peroxy radicals with NO_2_ and NO, respectively. More intense indoor lighting (UFT and FT) leads to higher concentrations of RO_2_ through additional reactions of OH with VOCs, although NO and NO_2_ concentrations are suppressed as explained above. However, overall, the higher RO_2_ concentrations produce more PANs and organic nitrates for these lighting conditions compared to the others.

In summary, the differences between indoor lights are relatively modest, although there clearly are some differences, with some of the more intense fluorescent lights generating higher OH concentrations than the other lighting we simulated.

### 3.4 Impacts of glass type on indoor air chemistry

**FIGURE 3:**
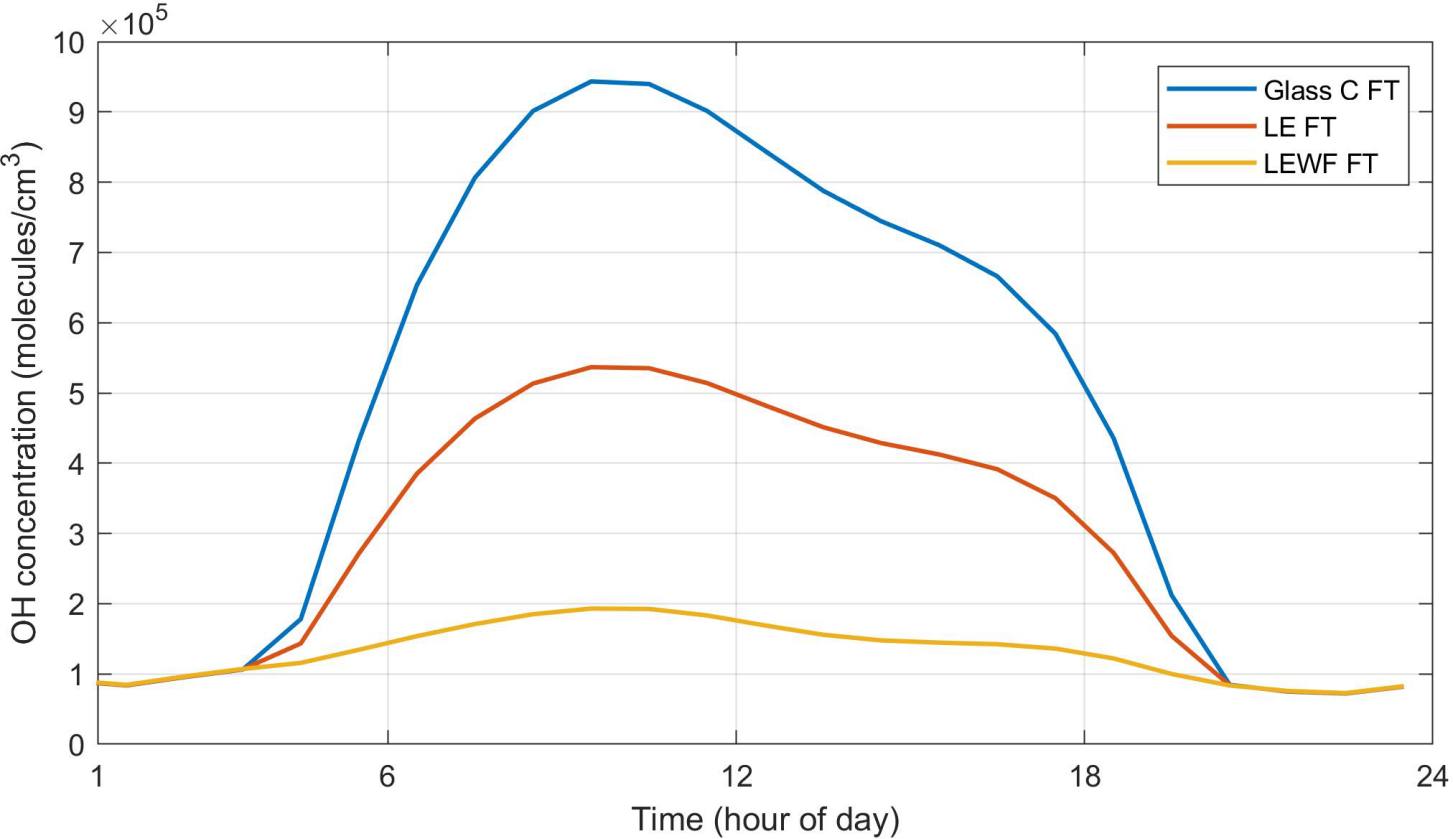
Hourly averaged concentrations of OH assuming FT with Glass C, LE, or LEWF

Figure 3 shows predicted OH concentrations for the case with indoor fluorescent tube lighting (FT) coupled with attenuated sunlight passing through three different glass types (Glass C, LE, and LEWF). The average values (between 06:00 and 18:00 h) for all studied key species in each model run are summarized in Table S5, along with the results from the other fluorescent tube lights (UFT and CFT).

For O_3_, HONO, NO_2,_ and OH, the impact of different glasses is very clear and much more important than changes in artificial lighting sources. Peak RO_2_ concentrations are highest for LEWF, corresponding to the lowest peak concentrations of NO: higher NO concentrations suppress those of RO_2_. For HO_2_, peak concentrations are highest under UFT for LEWF glass, followed by Glass C and LE, while its peak concentrations under CFT and FT are highest for Glass C, followed by LEWF and LE. Although OH reactions can form HO_2_ and RO_2_ through reactions with VOCs, NO reacts rapidly with the peroxy radicals once formed. Peak NO concentrations are very low under LEWF, approximately 2 and 1.6 times lower than the concentration under Glass C and LE, respectively, so less NO is available to react with HO_2_ and RO_2_. In fact, increased photolysis rates will increase OH concentrations and hence its ability to form HO_2_ and RO_2_, but also form more NO from NO_2_ photolysis under the same conditions, which can then react with HO_2_ and RO_2_. There are subtle differences in the balance between these processes which determine the predicted peroxy radical concentrations.

### 3.5 Impact of cloudiness on indoor air chemistry

**FIGURE 4:**
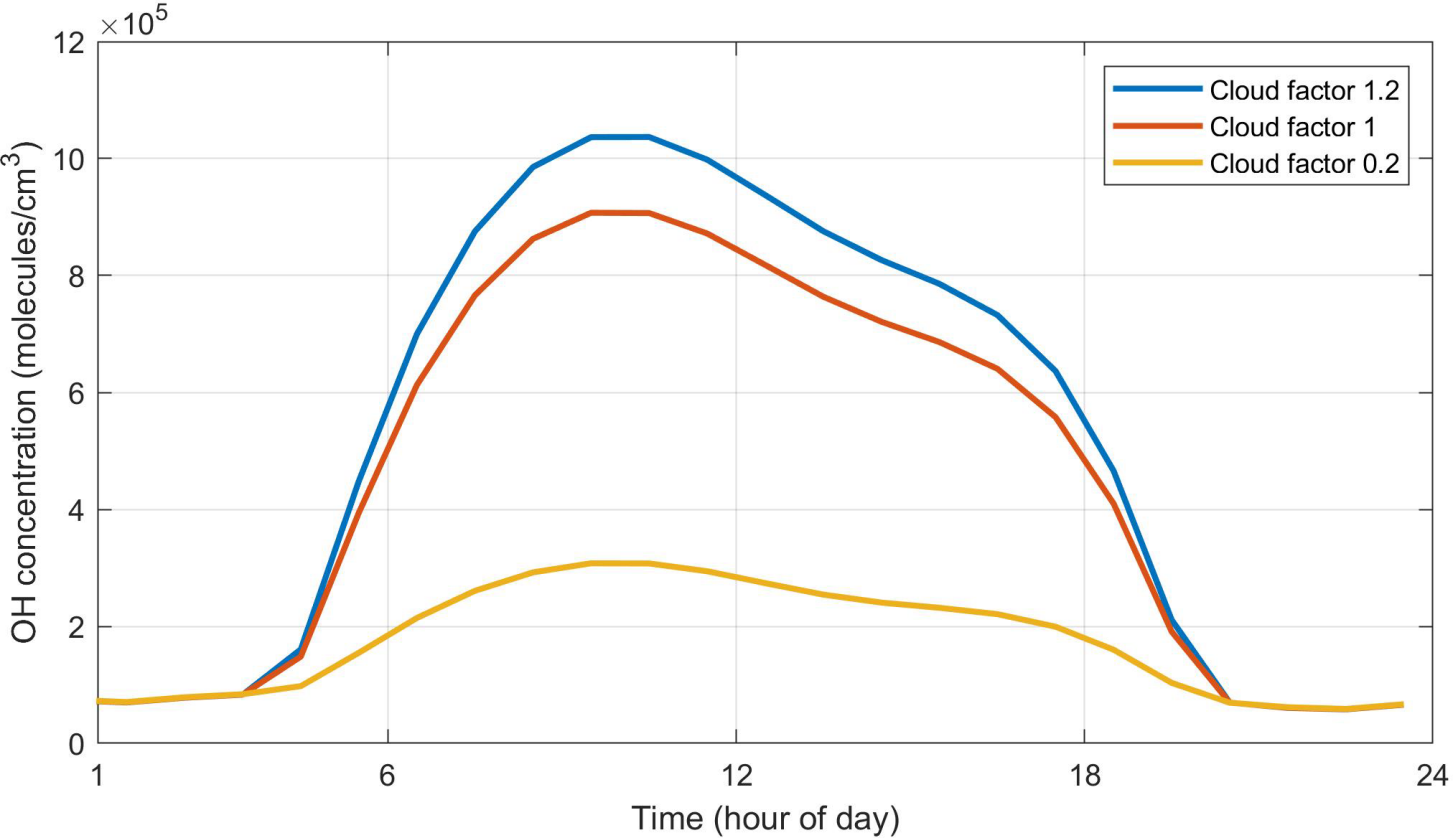
Hourly averaged concentrations of OH for cloudiness factors of 0.2, 1, and 1.2 and for LE glass and no indoor lighting

The level of cloudiness outdoors can have a large impact on predicted indoor concentrations of different species. Crawford^10^ investigated the impacts of clouds on spectral actinic flux at the Earth's surface and found that increased cloud fraction could enhance or decrease the surface actinic flux, depending on the cloud conditions. The greatest enhancement (a factor of 1.2 compared to clear sky) happened with an unoccluded solar disk and slightly overcast conditions, while the highest reduction (a factor of 0.2 compared to clear sky) took place with an occluded solar disk and more overcast conditions. Figure 4 shows an example for predicted OH concentrations for cloudiness factors of 0.2, 1, and 1.2 and for LE glass and no indoor lighting, while Table S6 summarizes the average indoor concentrations for each of the key model species for each of the model runs between 06:00 and 18:00 h. The reduction in [OH] of 66% going from cloudiness factors of 1.0–0.20 is in good agreement with a predicted [OH] reduction of 60%–80% under cloudy conditions compared to sunny conditions, based on measured photon fluxes.^4^

The concentrations of O_3_, HCHO, OH, NO, TOTPAN, and TOTORGNO3 increase as the cloudiness factor increases, while HONO and NO_2_ concentrations decrease. The changes in RO_2_ and HO_2_ concentrations are again more complex in terms of their relationship with different cloud levels. Again, this is due to the interplay between increased light leading to more OH radicals and hence more oxidation of VOCs to produce peroxy (HO_2_ and RO_2_) radicals, versus more NO produced from photolysis that removes the same peroxy radicals.

These results show that assuming clear sky conditions may mean that predicted indoor concentrations could be over or underestimated, which could have potentially greater impacts in some locations compared to others. For instance, the annual average sunshine duration in Marsa Alam, Egypt (25°N) is 3958 hours, compared to 1203 hours in Glasgow, United Kingdom (56°N).^45^ Assuming clear sky conditions may be reasonable for Marsa Alam, but less so for Glasgow, with consequential impacts on model predictions.

### 3.6 Impact of time of year and latitude on indoor air chemistry

The model runs in local solar time, with the time of year, day, and the latitude determining the location of the sun in the sky for each model run. For the simulations investigating the impact of the time of year and variation in latitude on indoor photolysis rates, indoor lighting was assumed to be off and indoor light was from attenuated sunlight only via Glass C, with a cloudiness factor of 1. One day from each month (21st) was then simulated to study the impacts of different times of year on indoor air chemistry, and we also ran simulations for the Equator (0°), 10°, 20°, 30°, 40°, 50°, 60°, and 65°N.

**FIGURE 5:**
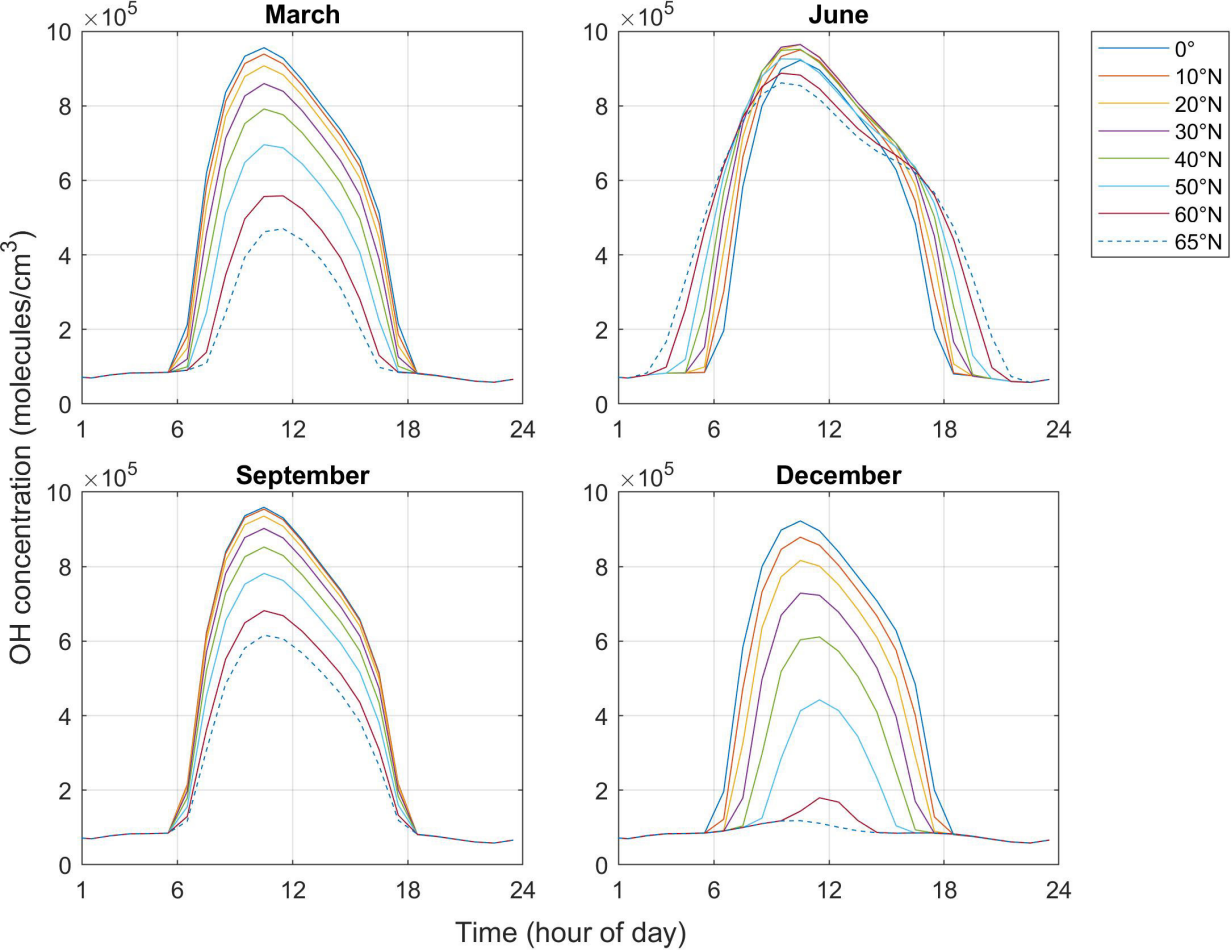
Diurnal profiles of hourly averaged OH concentrations at the Equator (0°), 10°N, 20°N, 30°N, 40°N, 50°N, 60°N, and 65°N assuming no internal lighting and glass C on the 21st of March, June, September, and December

Figure 5 shows the concentrations of OH for latitudes between 0° and 65°N and for March, June, September, and December 21. Table S7 shows the average concentration for all of the studied species between 06:00 and 18:00 h. The differences in average concentrations during the year are small at the Equator, becoming much greater as latitude increases.

In spring, autumn, and winter, the maximum OH concentrations decrease as the latitude increases, owing to more intense sunlight at the Equator. In summer, more sunlight reaches the Earth's surface over the entire hemisphere, leading to smaller differences in concentrations of key indoor species with latitude compared to the other seasons. In addition, the daylight hours during summer increase with latitude. Because of the interplay between these two factors, the average concentrations of OH are highest at mid‐latitudes (between 30°N and 40°N). Although sunlight is not as intense at these latitudes as at the Equator, there are more daylight hours, such that overall photolysis rates peak.

The HCHO and NO_2_ concentrations are relatively invariant throughout these simulations, suggesting that indoor photolysis does not control their concentrations. Another four sensitivity tests were carried out, in which the deposition rate, air exchange rate, outdoor HCHO concentration, or outdoor NO_2_ concentration were doubled. For HCHO, the results show that doubling the deposition rate, air exchange rate or outdoor HCHO concentration caused the average HCHO concentration (between 06:00 and 18:00 h) to decrease by 45% and 13% and increase by 2%, respectively, compared to the baseline condition. For NO_2_, the results show that doubling the deposition rate, air exchange rate or outdoor NO_2_ concentration caused the average NO_2_ concentration (between 06:00 and 18:00 h) to decrease by 45% and increase by 68% and 193%, respectively, compared to the baseline. Therefore, both HCHO and NO_2_ concentrations are clearly controlled by factors other than photolysis.

### 3.7 Changes over time

A final model test was run to consider how changes in lighting over time could impact indoor air chemistry. The formation of fine particulate matter (PM_2.5_) following cleaning was investigated under different indoor lighting conditions and glass types broadly representative of different points in time. For Run 1, it was assumed that there was incandescent lighting indoors, combined with Glass‐C. Glass C transmits light above 315 nm, and we assume this to be broadly representative of the single pane, simple composition glasses that were more common in UK homes before energy efficiency measures were introduced. For Run 2, it was assumed that LEDs were used with a lower light transmission glass (LEWF), as a proxy for more modern housing. For both runs, it was assumed there was a cleaning event (the emissions were assumed to last for one hour) with the use of a limonene‐containing cleaning product, based on the conditions described in Carslaw et al.^25^. Peak mixing ratios of limonene were ~170–180 ppb for both runs.

The peak PM_2.5_ concentration for Run 1 was 138 µg/m^3^, approximately 40% higher than the peak concentration for Run 2 of 98 µg/m^3^. Both runs had background PM_2.5_ concentrations of approximately 10 µg/m^3^ before cleaning started. Therefore, it is likely that our assumed modern lighting and window conditions have reduced indoor photolysis rates and hence the production of secondary pollutants such as PM_2.5_, all other things (ventilation rate, outdoor pollutant concentrations, indoor emissions, etc.) being equal.

## 4 CONCLUSION

This study has shown that indoor photolysis can play an important role in indoor air chemistry and hence indoor air quality. Many factors influence the role of indoor photolysis on indoor air chemistry, including indoor artificial light type, glass composition, degree of cloudiness, time of year, and location. Removal by photolysis can be more important than via air exchange with outdoors for some species. For instance, for an air exchange rate of 0.76 h^−1^ and assuming we have Glass C and incandescent lighting, removal by photolysis is more important for HONO, NO_2,_ and NO_3_ than through air exchange. For an air exchange rate of 0.2 h^−1^, photolysis of some of the carbonyl species also starts to out compete removal via air exchange.

For future studies, more measurements of indoor photolysis rates are essential, as well as measurements of the key species focused on in this study for different indoor lighting levels. In addition, it would be helpful for modeling studies if measurements of other species such as acetaldehyde and PM were made under different indoor lighting conditions. This information could possibly be gained through use of a sensor network, where sensors measuring different chemical species and photon intensities could be placed in different places around a room/building under different lighting conditions (e.g., dark, attenuated sunlight only, indoor artificial lights only and so on) and at different times of day. The collected data can then be used in a model to simulate the radical concentrations and further understand the impacts.

Finally, it is worth reiterating that health data for indoor (and outdoor) air pollutants is currently only available for relatively few species. This limits the ability to fully quantify the impacts of reducing indoor photolysis on human health. Future studies should focus on assessing which type of lighting indoors (combination of glass type and artificial lighting) is most beneficial for human health, through testing the health effects of the different air pollutant mixtures formed under the different lighting conditions that are typically experienced in residences and other buildings.

## AUTHOR CONTRIBUTION

Zixu Wang: Methodology, Software, Formal analysis, Validation, Investigation, Writing – Original Draft. David Shaw: Software, Investigation, Data curation, Writing – Review and Editing, Visualization. Tara Kahan: Provision of experimental data (artificial lighting photon flux intensities), Validation, Writing – Review and Editing. Coralie Schoemaecker: Provision of data (attenuated photon flux intensities for different glass materials), Writing – Review and Editing. Nicola Carslaw: Conceptualisation, Methodology, Software, Supervision, Writing – Original Draft, Writing – Review and Editing, Project administration, Funding acquisition.

### PEER REVIEW

The peer review history for this article is available at https://publons.com/publon/10.1111/ina.13054.

## Supporting information

Table S1‐S7

## Data Availability

Data from this project are available on Pure, an online data repository hosted by the University of York.^46^
