# Supplementary material for: A modeling study of the impact of photolysis on indoor air quality
Source: Indoor Air. 2022 Jun 13;32(6):e13054. doi: 10.1111/ina.13054 (PMC9328129; doi:10.1111/ina.13054)
Supplement: Supplementary file 1 — Table S1‐S7 [file INA-32-0-s001.docx]

Supplementary Information

***Table S1***: Wavelength range and interval for provided absorption cross section and quantum yield data for each of the species/groups of species undergoing photolysis. Where more than one set of products is possible, the main product is shown in brackets. The absorption cross section and quantum yield data are taken from IUPAC (IUPAC, nd) or the MCM (Jenkin et al., 1997; Saunders et al., 2003). The *j* value labels are according to the convention used in the MCM.

| J value | Species | Wavelengths range (nm) | Wavelength interval (nm) |
| --- | --- | --- | --- |
| J1 | O_3_ (O(1D)) | 300-349 | 1 |
| J2 | O_3_ (O(^3^P)) | 300-349; 400-700 | 1 |
| J3 | H_2_O_2_ | 300-350 | 5 |
| J4 | NO_2_ | 300-425 | 5 |
| J5 | NO_3_ (NO + O_2_) | 586-640 | 1 |
| J6 | NO_3_ (NO_2_ + O(^3^P)) | 585-640 | 1 |
| J7 | HONO | 300-399 | 1 |
| J8 | HNO_3_ | 300-350 | 5 |
| J11 | HCHO (H+HCO) | 300-355 | 1 |
| J12 | HCHO (H_2_+CO) | 300-360 | 1 |
| J13 | CH_3_CHO | 300-330 | 5 |
| J14 | C_2_H_5_CHO | 300-330 | 5 |
| J15 | C_3_H_7_CHO (n-C_3_H_7_ + HCO) | 300-364 | 1 |
| J16 | C_3_H_7_CHO (C_2_H_4_ + CH_2_CHOH) | 300-364 | 1 |
| J17 | i-C_3_H_7_CHO | 300-330 | 5 |
| J18 | CH_2_C(CH_3_)CHO (CH_2_=CCH_3_+HCO) | 300-395 | 1 |
| J19 | CH_2_C(CH_3_)CHO (CH_2_C(CH_3_)CO+H) | 300-395 | 1 |
| J21 | CH_3_C(O)CH_3_ | 300-327 | 1 |
| J22 | CH_3_C(O)C_2_H_5_ (CH_3_CO+C_2_H_5_) | 300-352 | 1 |
| J23 | CH_3_C(O)CH=CH_2_ (CH_3_CH=CH_2_ + CO) | 300-395 | 1 |
| J24 | CH_3_C(O)CH=CH_2_ (CH_3_CO + CH_2_=CH) | 300-395 | 1 |
| J31 | CHOCHO (CO + CO + H_2_) | 300-355 | 5 |
| J32 | CHOCHO (HCHO + CO) | 300-415 | 5 |
| J33 | CHOCHO (HCO + HCO) | 300-445 | 5 |
| J34 | CH_3_COCHO | 300-440 | 10 |
| J35 | CH_3_C(O)C(O)CH_3_ | 300-460 | 1 |
| J41 | CH_3_OOH | 300-365 | 5 |
| J51 | CH_3_ONO_2_ | 300-340 | 5 |
| J52 | C_2_H_5_ONO_2_ | 300-340 | 5 |
| J53 | n-C_3_H_7_ONO_2_ | 300-340 | 5 |
| J54 | (CH_3_)_2_CHONO_2_ | 300-360 | 5 |
| J55 | (CH_3_)_3_CONO_2_ | 300-330 | 5 |
| J56 | NOA (CH_3_C(O)CH_2_(O.) + NO_2_) | 300-340 | 5 |
| J57 | NOA (CH_3_CO + HCHO + NO_2_) | 300-340 | 5 |

***Table S2***: Calculated photolysis coefficients (in units of s^-1^) for the 35 species and for 7 different indoor artificial lights (1m away from the light sources).

| J value | | Incand. | Halogen | LED | | CFL | | UFT | | CFT | | | FT |
| --- | --- | --- | --- | --- | --- | --- | --- | --- | --- | --- | --- | --- | --- |
| J1 | | 3.6×10^-8^ | 1.2×10^-8^ | 0 | | 9.6×10^-10^ | | 1.4×10^-6^ | | 4.8×10^-12^ | | | 4.1×10^-7^ |
| J2 | | 4.3×10^-6^ | 3.7×10^-6^ | 4.3×10^-6^ | | 6.1×10^-6^ | | 1.0×10^-5^ | | 4.0×10^-6^ | | | 3.3×10^-6^ |
| J3 | | 4.5×10^-9^ | 4.0×10^-9^ | 3.6×10^-10^ | | 3.6×10^-9^ | | 1.7×10^-7^ | | 6.3×10^-10^ | | | 5.1×10^-8^ |
| J4 | | 1.1×10^-5^ | 1.3×10^-5^ | 5.0×10^-7^ | | 4.5×10^-5^ | | 5.7×10^-5^ | | 6.5×10^-6^ | | | 2.0×10^-5^ |
| J5 | | 3.7×10^-4^ | 3.2×10^-4^ | 5.5×10^-4^ | | 9.5×10^-4^ | | 1.3×10^-3^ | | 5.6×10^-4^ | | | 4.1×10^-4^ |
| J6 | | 1.4×10^-3^ | 1.2×10^-3^ | 2.0×10^-3^ | | 3.4×10^-3^ | | 4.9×10^-3^ | | 2.3×10^-3^ | | | 1.6×10^-3^ |
| J7 | | 1.4×10^-6^ | 1.8×10^-6^ | 0 | | 6.3×10^-6^ | | 1.0×10^-5^ | | 1.5×10^-7^ | | | 3.5×10^-6^ |
| J8 | | 5.3×10^-10^ | 2.8×10^-10^ | 0 | | 5.5×10^-11^ | | 2.6×10^-8^ | | 0 | | | 7.4×10^-9^ |
| J11 | | 1.5×10^-8^ | 1.2×10^-8^ | 0 | | 2.9×10^-9^ | | 7.8×10^-7^ | | 0 | | | 2.3×10^-7^ |
| J12 | | 2.1×10^-8^ | 2.3×10^-8^ | 0 | | 2.2×10^-8^ | | 6.5×10^-7^ | | 0 | | | 2.0×10^-7^ |
| J13 | | 4.3×10^-9^ | 2.2×10^-9^ | 0 | | 3.7×10^-11^ | | 2.3×10^-7^ | | 0 | | | 6.6×10^-8^ |
| J14 | | 1.1×10^-8^ | 5.0×10^-9^ | 0 | | 7.1×10^-11^ | | 6.3×10^-7^ | | 0 | | | 1.8×10^-7^ |
| J15 | | 6.0×10^-9^ | 4.6×10^-9^ | 0 | | 1.6×10^-9^ | | 3.0×10^-7^ | | 8.8×10^-13^ | | | 8.7×10^-8^ |
| J16 | | 2.8×10^-9^ | 2.2×10^-9^ | 0 | | 7.7×10^-10^ | | 1.4×10^-7^ | | 4.2×10^-13^ | | | 4.1×10^-8^ |
| J17 | | 3.2×10^-8^ | 2.4×10^-8^ | 0 | | 4.6×10^-9^ | | 1.7×10^-6^ | | 0 | | | 4.8×10^-7^ |
| J18 | | 6.1×10^-10^ | 7.2×10^-10^ | 0 | | 2.7×10^-9^ | | 1.1×10^-8^ | | 4.3×10^-11^ | | | 3.6×10^-9^ |
| J19 | 6.1×10^-10^ | | 7.2×10^-10^ | | 0 | | 2.7×10^-9^ | | 1.1×10^-8^ | | 4.3×10^-11^ | 3.6×10^-9^ | |
| J21 | 3.7×10^-10^ | | 1.1×10^-10^ | | 0 | | 0 | | 1.6×10^-8^ | | 0 | 4.5×10^-9^ | |
| J22 | 1.1×10^-9^ | | 5.9×10^-10^ | | 0 | | 3.4×10^-11^ | | 6.1×10^-8^ | | 0 | 1.7×10^-8^ | |
| J23 | 1.1×10^-9^ | | 1.0×10^-9^ | | 0 | | 1.7×10^-9^ | | 3.9×10^-8^ | | 1.8×10^-11^ | 1.2×10^-8^ | |
| J24 | 1.1×10^-9^ | | 1.0×10^-9^ | | 0 | | 1.7×10^-9^ | | 3.9×10^-8^ | | 1.8×10^-11^ | 1.2×10^-8^ | |
| J31 | 3.8×10^-9^ | | 3.0×10^-9^ | | 0 | | 1.6×10^-9^ | | 1.7×10^-7^ | | 0 | 5.1×10^-8^ | |
| J32 | 1.9×10^-8^ | | 1.8×10^-8^ | | 1.4×10^-10^ | | 3.8×10^-8^ | | 6.4×10^-7^ | | 4.3×10^-9^ | 1.9×10^-7^ | |
| J33 | 7.5×10^-8^ | | 8.4×10^-8^ | | 3.2×10^-8^ | | 3.5×10^-7^ | | 1.1×10^-6^ | | 1.3×10^-7^ | 3.3×10^-7^ | |
| J34 | 1.3×10^-7^ | | 1.4×10^-7^ | | 2.6×10^-8^ | | 5.9×10^-7^ | | 2.2×10^-6^ | | 1.7×10^-7^ | 6.8×10^-7^ | |
| J35 | 5.4×10^-7^ | | 6.0×10^-7^ | | 7.7×10^-7^ | | 2.4×10^-6^ | | 3.7×10^-6^ | | 1.5×10^-6^ | 1.2×10^-6^ | |
| J41 | 3.1×10^-9^ | | 2.9×10^-9^ | | 0 | | 7.4×10^-9^ | | 1.1×10^-7^ | | 8.4×10^-11^ | 3.3×10^-8^ | |
| J51 | 8.0×10^-10^ | | 4.3×10^-10^ | | 0 | | 6.2×10^-11^ | | 4.1×10^-8^ | | 0 | 1.2×10^-8^ | |
| J52 | 1.2×10^-9^ | | 6.7×10^-10^ | | 0 | | 1.2×10^-10^ | | 6.1×10^-8^ | | 0 | 1.8×10^-8^ | |
| J53 | 1.4×10^-9^ | | 8.7×10^-10^ | | 0 | | 3.8×10^-10^ | | 6.6×10^-8^ | | 0 | 1.9×10^-8^ | |
| J54 | 2.0×10^-9^ | | 1.1×10^-9^ | | 0 | | 3.0×10^-10^ | | 9.6×10^-8^ | | 5.4×10^-13^ | 2.8×10^-8^ | |
| J55 | 4.5×10^-9^ | | 2.9×10^-9^ | | 0 | | 4.6×10^-10^ | | 2.3×10^-7^ | | 0 | 6.8×10^-8^ | |
| J56 | 1.8×10^-8^ | | 1.3×10^-8^ | | 0 | | 4.7×10^-9^ | | 8.7×10^-7^ | | 0 | 2.5×10^-7^ | |
| J57 | 1.8×10^-8^ | | 1.3×10^-8^ | | 0 | | 4.7×10^-9^ | | 8.7×10^-7^ | | 0 | 2.5×10^-7^ | |

***Table S3***: Example of calculation of transmission factor for NO_2_ photolysis with Glass C in the windows.

| wavelength/nm | σ x ϕ (nm^2^ molecule^-1^) | % contribution to total for each specific wavelength | % transmission factor (Glass C) from Blocquet et al. (2018) | Weighted transmission factor |
| --- | --- | --- | --- | --- |
| 300 | 1.3×10^-19^ | 1.4 | 0 | 0 |
| 305 | 1.6×10^-19^ | 1.7 | 0 | 0 |
| 310 | 1.9×10^-19^ | 2.0 | 0 | 0 |
| 315 | 2.2×10^-19^ | 2.4 | 0.8 | 0.02 |
| 320 | 2.5×10^-19^ | 2.8 | 4.3 | 0.1 |
| 325 | 2.9×10^-19^ | 3.1 | 7.7 | 0.2 |
| 330 | 3.2×10^-19^ | 3.5 | 18.7 | 0.7 |
| 335 | 3.6×10^-19^ | 3.9 | 26.6 | 1.0 |
| 340 | 4.0×10^-19^ | 4.4 | 42.1 | 1.8 |
| 345 | 4.2×10^-19^ | 4.5 | 53.1 | 2.4 |
| 350 | 4.6×10^-19^ | 5.0 | 61.9 | 3.1 |
| 355 | 5.0×10^-19^ | 5.4 | 66.0 | 3.6 |
| 360 | 5.1×10^-19^ | 5.5 | 70.1 | 3.9 |
| 365 | 5.5×10^-19^ | 6.0 | 73.9 | 4.4 |
| 370 | 5.6×10^-19^ | 6.1 | 77.6 | 4.7 |
| 375 | 5.9×10^-19^ | 6.4 | 77.6 | 5.0 |
| 380 | 5.9×10^-19^ | 6.4 | 77.6 | 5.0 |
| 385 | 5.9×10^-19^ | 6.5 | 79.7 | 5.1 |
| 390 | 6.2×10^-19^ | 6.7 | 81.7 | 5.5 |
| 395 | 5.9×10^-19^ | 6.4 | 81.7 | 5.3 |
| 400 | 5.6×10^-19^ | 6.1 | 81.7 | 5.0 |
| 405 | 2.1×10^-19^ | 2.3 | 81.7 | 1.9 |
| 410 | 9.2×10^-20^ | 1.0 | 82.1 | 0.8 |
| 415 | 3.5×10^-20^ | 0.4 | 82.4 | 0.3 |
| 420 | 1.1×10^-20^ | 0.1 | 82.4 | 0.1 |
| 425 | 2.3×10^-21^ | 0 | 82.4 | 0 |
| Sum of (σ x ϕ) = 9.2×10^-18^ | |  | Sum of weighted transmission = 59.9% | |

***Table S4***: Calculated transmission factors (%/100) for 35 species/groups of species that undergo photolysis and for the three window glasses studied. The absorption cross section and quantum yield data are taken from IUPAC (IUPAC, nd) or the MCM (Jenkin et al., 1997; Saunders et al., 2003).

|  | Glass C | Low Emissivity | Low Emissivity with film |
| --- | --- | --- | --- |
| J1 | 0.003 | 0.0001 | 0 |
| J2 | 0.17 | 0.1 | 0.08 |
| J3 | 0.06 | 0.01 | 0 |
| J4 | 0.6 | 0.3 | 0.04 |
| J5 | 0.85 | 0.63 | 0.51 |
| J6 | 0.85 | 0.66 | 0.55 |
| J7 | 0.58 | 0.24 | 0.001 |
| J8 | 0.01 | 0.0004 | 0 |
| J11 | 0.03 | 0.001 | 0 |
| J12 | 0.15 | 0.02 | 0 |
| J13 | 0.003 | 0 | 0 |
| J14 | 0.001 | 0 | 0 |
| J15 | 0.03 | 0.002 | 0 |
| J16 | 0.03 | 0.002 | 0 |
| J17 | 0.02 | 0 | 0 |
| J18 | 0.3 | 0.09 | 0.0001 |
| J19 | 0.3 | 0.09 | 0.0001 |
| J20 | 0.3 | 0.09 | 0.0001 |
| J21 | 0.001 | 0 | 0 |
| J22 | 0.01 | 0.0002 | 0 |
| J23 | 0.09 | 0.02 | 0 |
| J24 | 0.09 | 0.02 | 0 |
| J31 | 0.03 | 0.003 | 0 |
| J32 | 0.07 | 0.02 | 0.002 |
| J33 | 0.25 | 0.11 | 0.04 |
| J34 | 0.16 | 0.08 | 0.02 |
| J35 | 0.77 | 0.51 | 0.34 |
| J41 | 0.08 | 0.02 | 0 |
| J51 | 0.01 | 0.0001 | 0 |
| J52 | 0.01 | 0.0002 | 0 |
| J53 | 0.02 | 0.001 | 0 |
| J54 | 0.01 | 0.001 | 0 |
| J55 | 0.01 | 0 | 0 |
| J56 | 0.02 | 0.001 | 0 |
| J57 | 0.02 | 0.001 | 0 |

***Table S5***: Average concentrations of O_3_, HONO, NO_2_, OH, HO_2_, RO_2_, NO, TOTPAN and TOTORGNO_3_ for UFT, CFT and FT of Glass C, LE and LEWF over the model simulation between 06:00-18:00 h. O_3_, NO and NO_2_ in unit of ppb; HONO, HO_2_, RO_2_, TOTPAN and TOTORGNO_3_ in unit of ppt; OH in unit of 10^5^ molecule/cm^3^.

|  | Glass C | | | LE | | | LEWF | | |
| --- | --- | --- | --- | --- | --- | --- | --- | --- | --- |
|  | UFT | FT | CFT | UFT | FT | CFT | UFT | FT | CFT |
| O_3_ | 8.1 | 7.8 | 7.7 | 6.3 | 6.1 | 6.0 | 4.5 | 4.4 | 4.3 |
| HONO | 157.8 | 159.1 | 159.6 | 195.4 | 197.2 | 197.8 | 241.3 | 243.9 | 245.0 |
| NO_2_ | 3.0 | 3.0 | 3.1 | 3.2 | 3.2 | 3.3 | 3.5 | 3.5 | 3.5 |
| OH | 8.6 | 7.9 | 7.6 | 5.2 | 4.6 | 4.3 | 2.0 | 1.6 | 1.5 |
| HO_2_ | 5.8 | 4.9 | 4.6 | 4.9 | 4.0 | 3.6 | 5.6 | 4.1 | 3.5 |
| RO_2_ | 6.6 | 5.8 | 5.5 | 5.9 | 5.0 | 4.7 | 7.6 | 6.4 | 5.8 |
| NO | 2.3 | 2.5 | 2.6 | 1.7 | 1.9 | 2.0 | 0.7 | 0.9 | 0.9 |
| TOTPAN | 390.7 | 347.2 | 329.2 | 319.7 | 276.2 | 258.1 | 243.2 | 207.1 | 191.4 |
| TOTORGNO_3_ | 181.8 | 171.3 | 166.6 | 117.3 | 107.2 | 102.7 | 48.6 | 41.7 | 38.5 |

***Table S6*:** Average concentrations of O_3_, HONO, NO_2_, OH, HO_2_, RO_2_, NO, TOTPAN and TOTORGNO_3_ for cloud 1.2, 1 and 0.2 for the LE Glass and no indoor lights between 06:00-18:00h. O_3_, NO and NO_2_ are in units of ppb; HONO, HO_2_ and RO_2_, TOTPAN and TOTORGNO_3_ in units of ppt; OH in units of 10^5^ molecule/cm^3^.

|  | O_3_ | HONO | NO_2_ | OH | HO_2_ | RO_2_ | NO | TOTPAN | TOTORGNO_3_ |
| --- | --- | --- | --- | --- | --- | --- | --- | --- | --- |
| CF 0.2 | 4.9 | 221.7 | 3.4 | 2.6 | 3.7 | 5.2 | 1.3 | 235.2 | 63.8 |
| CF 1 | 7.7 | 159.6 | 3.1 | 7.6 | 4.6 | 5.5 | 2.6 | 329.2 | 166.4 |
| CF 1.2 | 8.2 | 149.8 | 3.0 | 8.7 | 4.8 | 5.6 | 2.8 | 344.0 | 186.3 |

***Table S7***: Average concentrations of key output species between 0˚ and 65˚N for different latitudes and for four seasons between 06:00-18:00 h. O_3_, NO and NO_2_ are in units of ppb; HONO, HO_2_ and RO_2_, in units of ppt; OH in units of 10^5^ molecule/cm^3^.

|  |  | O_3_ | HONO | HCHO | OH | HO_2_ | RO_2_ | NO | NO_2_ |
| --- | --- | --- | --- | --- | --- | --- | --- | --- | --- |
| March 21^st^ | 0˚ | 7.3 | 173.2 | 33.3 | 6.9 | 4.4 | 5.4 | 2.3 | 3.1 |
|  | 10˚N | 7.2 | 176.5 | 33.3 | 6.7 | 4.4 | 5.4 | 2.3 | 3.1 |
|  | 20˚N | 7.0 | 181.3 | 33.3 | 6.3 | 4.3 | 5.3 | 2.2 | 3.2 |
|  | 30˚N | 6.7 | 188.0 | 33.3 | 5.9 | 4.2 | 5.3 | 2.1 | 3.2 |
|  | 40˚N | 6.4 | 197.1 | 33.3 | 5.3 | 4.1 | 5.2 | 2.0 | 3.2 |
|  | 50˚N | 6.0 | 209.7 | 33.2 | 4.4 | 3.9 | 5.2 | 1.8 | 3.3 |
|  | 60˚N | 5.4 | 227.3 | 33.2 | 3.4 | 3.7 | 5.4 | 1.5 | 3.4 |
|  | 65˚N | 5.0 | 238.6 | 33.2 | 2.7 | 3.7 | 5.5 | 1.3 | 3.4 |
| June 21^st^ | 0˚ | 7.1 | 176.9 | 33.3 | 6.6 | 4.4 | 5.3 | 2.3 | 3.1 |
|  | 10˚N | 7.4 | 169.2 | 33.3 | 7.1 | 4.5 | 5.4 | 2.4 | 3.1 |
|  | 20˚N | 7.6 | 163.3 | 33.3 | 7.4 | 4.6 | 5.5 | 2.5 | 3.1 |
|  | 30˚N | 7.7 | 159.8 | 33.3 | 7.7 | 4.6 | 5.5 | 2.6 | 3.1 |
|  | 40˚N | 7.7 | 158.4 | 33.3 | 7.7 | 4.6 | 5.5 | 2.6 | 3.0 |
|  | 50˚N | 7.7 | 158.9 | 33.3 | 7.7 | 4.6 | 5.5 | 2.6 | 3.1 |
|  | 60˚N | 7.6 | 161.1 | 33.3 | 7.5 | 4.5 | 5.4 | 2.6 | 3.1 |
|  | 65˚N | 7.5 | 163.0 | 33.3 | 7.3 | 4.5 | 5.4 | 2.6 | 3.1 |
| September 21^st^ | 0˚ | 7.3 | 172.9 | 33.3 | 6.9 | 4.4 | 5.4 | 2.3 | 3.1 |
|  | 10˚N | 7.3 | 173.2 | 33.3 | 6.9 | 4.4 | 5.4 | 2.3 | 3.1 |
|  | 20˚N | 7.2 | 175.1 | 33.3 | 6.7 | 4.4 | 5.4 | 2.3 | 3.1 |
|  | 30˚N | 7.1 | 178.6 | 33.3 | 6.5 | 4.3 | 5.3 | 2.3 | 3.1 |
|  | 40˚N | 6.8 | 184.1 | 33.3 | 6.1 | 4.2 | 5.2 | 2.2 | 3.2 |
|  | 50˚N | 6.5 | 192.2 | 33.3 | 5.5 | 4.1 | 5.1 | 2.1 | 3.2 |
|  | 60˚N | 6.1 | 204.2 | 33.2 | 4.7 | 3.9 | 5.1 | 1.9 | 3.3 |
|  | 65˚N | 5.8 | 212.3 | 33.2 | 4.2 | 3.8 | 5.0 | 1.7 | 3.3 |
| December 21^st^ | 0˚ | 7.1 | 176.9 | 33.3 | 6.6 | 4.4 | 5.3 | 2.3 | 3.1 |
|  | 10˚N | 6.8 | 186.1 | 33.3 | 6.0 | 4.2 | 5.3 | 2.1 | 3.2 |
|  | 20˚N | 6.4 | 197.0 | 33.3 | 5.3 | 4.1 | 5.3 | 2.0 | 3.2 |
|  | 30˚N | 6.0 | 210.4 | 33.2 | 4.5 | 3.9 | 5.4 | 1.7 | 3.3 |
|  | 40˚N | 5.4 | 227.2 | 33.2 | 3.5 | 3.8 | 5.5 | 1.4 | 3.4 |
|  | 50˚N | 4.7 | 248.0 | 33.1 | 2.3 | 3.7 | 6.1 | 1.1 | 3.5 |
|  | 60˚N | 4.1 | 269.4 | 33.1 | 1.1 | 3.6 | 7.5 | 0.7 | 3.6 |
|  | 65˚N | 4.0 | 272.5 | 33.1 | 1.0 | 3.7 | 8.2 | 0.7 | 3.6 |

**References**

IUPAC (nd). Available online: <https://iupac-aeris.ipsl.fr/>

Jenkin, M. E.; Saunders, S. M.; Pilling, M. J. The tropospheric degradation of volatile organic compounds: a protocol for mechanism development. Atmospheric Environment. 1997, 31, 81-104. DOI: 10.1016/S1352-2310(96)00105-7.

MCM (nd). Available online: <http://mcm.york.ac.uk/MCM/parameters/photolysis.htt>

Saunders, S. M.; Jenkin, M. E.; Derwent, R. G.; Pilling, M. J. Protocol for the development of the Master Chemical Mechanism, MCM v3 (Part A): tropospheric degradation of non-aromatic volatile organic compounds. Atmospheric Chemistry and Physics. 2003, 3, 161-180. DOI: 10.5194/acp-3-161-2003.
